# Production of Active Nonglycosylated Recombinant B-Chain of Type-2 Ribosome-Inactivating Protein from *Viscum articulatum* and Its Biological Effects on Peripheral Blood Mononuclear Cells

**DOI:** 10.1155/2011/283747

**Published:** 2011-03-30

**Authors:** Tzu-Li Lu, Jing-Yuan Chuang, Jai-Sing Yang, Shau-Ting Chiu, Nai-Wan Hsiao, Mei-Chen Wu, Shih-Hsiung Wu, Ching-Hsiang Hsu

**Affiliations:** ^1^Institute of Biochemical Sciences, National Taiwan University, Taipei 106, Taiwan; ^2^Institute of Biological Chemistry, Academia Sinica, Taipei 115, Taiwan; ^3^School of Medical Laboratory Science and Biotechnology, China Medical University, Taichung 404, Taiwan; ^4^School of Medicine, China Medical University, Taichung 404, Taiwan; ^5^Department of Botany, National Museum of Natural Science, Taichung 404, Taiwan; ^6^Institute of Biotechnology, National Changhua University of Education, Changhua 500, Taiwan; ^7^School of Chinese Medicine, China Medical University, Taichung 404, Taiwan

## Abstract

Type-2 ribosome-inactivating proteins, composed of a toxic A-chain and lectin-like B-chain, display various biological functions, including cytotoxicity and immunomodulation. We here cloned the lectin-like B-chain encoding fragment of a newly identified type-2 RIP gene, *articulatin* gene, from *Viscum articulatum*, into a bacterial expression vector to obtain nonglycosylated recombinant protein expressed in inclusion bodies. After purification and protein refolding, soluble refolded recombinant articulatin B-chain (rATB) showed lectin activity specific toward galactoside moiety and was stably maintained while stored in low ionic strength solution. Despite lacking glycosylation, rATB actively bound leukocytes with preferential binding to monocytes and *in vitro* stimulated PBMCs to release cytokines without obvious cytotoxicity. These results implicated such a B-chain fragment as a potential immunomodulator.

## 1. Introduction

Ribosome-inactivating proteins (RIPs; EC 3.2.2.22) are widespread among higher plants of different taxonomic origins and belong to a group of proteins that inhibit protein synthesis by enzymatically damaging ribosomes [1]. RIPs divide into structural families: type-1 single-chain proteins with *N*-*β*-glycosidase activity and the larger type-2 consisting of two distinct polypeptide chains. A-chain of type-2 shows the same enzymatic activity as type-1, selectively cleaving adenine from rRNA to interrupt interaction between elongation Factor II and ribosomes, hence terminating protein synthesis [2, 3]. Held together with A-chain by a disulfide bridge, B-chain structure is similar to lectin, specifically binding galactose, *N*-acetylgalactosamine, or *N*-acetylneuraminic acid. Binding lectin-like B-chain to carbohydrate moieties on the surface of most eukaryotic cells allows A-chain entry into cytoplasm, where it is enzymatically active [4].

Due to their unique structural and functional properties, some type-2 RIPs with high cytotoxicity have been implicated in cancer therapy and thoroughly examined for ability to kill cancer cells *in vitro* or *in vivo *for decades. One of the most noticeable is mistletoe lectin I (ML-I), which was isolated from* Viscum album *and has been identified as the main active component of mistletoe extracts, a widely applied herbal medicine in Europe for complementary treatment of cancer patients [5]. Interestingly, the possible antitumor effects of ML-I in animal models are based on its cytotoxic and immunomodulatory effects [6, 7]; it has also been considered as an immunomodulator. At subtoxic doses, ML-I, as well as ricin, has demonstrably enhanced release of cytokines from peripheral blood mononuclear cells [6, 8]. As ML-I is composed of an A-chain and a B-chain, clarifying the role of each subunit on immunostimulatory effect is needed for further research. Hajto et al. have demonstrated that the B-chain binding activity was necessary for immunostimulating effects induced by ML-I and that ML-I heterodimer as well as its isolated B-chain could stimulate immune cells to release cytokines [6]. Accordingly, it seemed that B-chain alone was sufficient to stimulate immune cells; however, higher isolated B-chain dose was needed to achieve the same effect as the ML-I heterodimer. This raises the question of whether isolated B-chain was in fact pure or contaminated with native heterodimer during its preparation process. Whether a B-chain fragment is an active immunomodulator thus remains questionable. A heterologous expression system, such as *E. coli*, can help obtain a pure and adequate amount of B-chain without heterodimer contamination. Successfully preparing such recombinant B-chain will facilitate probing of biological effects and potential use of the B-chain fragment.

Our previous study identified a new type-2 RIP gene from a Chinese mistletoe, *Viscum articulatum*, and designated it as the *articulatin* gene (GeneBank accession no. EF620539). This gene shares high sequence homology with ML-I gene. In this study, we expressed articulatin B-chain gene in *E. coli* and created the soluble rATB by an improved refolding protocol. We kept nonglycosylated rATB stable during storage for functioning and evaluated its biological effects on PBMCs.

## 2. Materials and Methods

### 2.1. Plant Material

*Viscum articulatum *Burm (Viscoidae, Loranthaceae *sensu lato*) was collected and authenticated by Dr. S.-T. Chiu at Bai Hua Ling, Bao Shan Shi, Yunnan Province, China (N 24°43′42.1′′, E 98°48′19.4′′) at an altitude of 2020 m in 2004. It invaded the stem of *Castanopsis* sp. The specimen (collection number S.-T. Chiu 08534) will be deposited at the herbarium (TNM) of the National Museum of Natural Science, Taichung, Taiwan.

### 2.2. Bacterial Strains and Plasmids

*Escherichia coli* XL1 blue (F^−^ *φ*80(*lacZ*)ΔM15 Δ*lacX74 hsdR*(*r_k_* ^−^, *m_k_* ^+^) Δ*recA1398 endA1 tonA*) and BL21 [F^−^ *ompT hsdSB*(*r_B_* ^−^ *m_B_* ^−^) *gal dcm* (DE3) pLysS(Cam^R^)] were obtained from Invitrogen. Plasmid pCR2.1-TOPO (Invitrogen) was used for cloning and sequencing, plasmid pET-28a(+) (Novagen) for expression.

### 2.3. Cloning and Preparation of Recombinant Articulatin B-Chain Expression Constructs

To clone the type-2 RIP gene from *Viscum articulatum*, we designed several primer sets based on the conserved nucleotide sequences within type-2 RIPs genes. Only one oligonucleotide primer set amplified a 919-bp DNA fragment of the gene encoding type-2 RIP by PCR (rAT3: 5′-CGCCATC GACGTTACCAATCTG-3′; rAT4: 5′-ATTGATGATGGTCCCATTGCCC-3′). The full-length gene was obtained by chromosome walking (CW) on *Viscum articulatum* genomic DNA using the LA PCR in vitro cloning kit (Takara, Japan) and the primary primers 5end-CW1 (5′-AGGGGCGTCGCGGAAAAAGTAGGATTG-3′) and 3end-CW1 (5′-GGGGCC AACAATCCACGCAAGTCCAGCAGT-3′), and the nested primers 5end-CW2 (5′-TCTGAATGGCGACTAAAAGGGAACGAGC-3′) and 3end-CW2 (5′-CAGCGA CCGGCCATCTTCCTCTTTGC-3′). The whole type-2 RIP gene from *Viscum articulatum* was designated as *articulatin* gene (GeneBank accession no. EF620539). Based on this information, we designed a specific pair of primers to amplify the *articulatin* B-chain gene nucleotide sequences: sense primer: rATB1 (5′-GGAATTC**CATATG **GACGATGTKACCTG CACTATTTCCG-3′) (*Nde*I site in bold); antisense primer: rATB2 (5′-ATCCG**CTCGAG** TTATTAAGGCACCGGAAG CCACATTTG-3′) (two stop codons, underlined; *Xho*I site in bold).

Amplified B-chain fragment was digested with *Nde*I and *Xho*I, then subcloned into pET-28a(+) to yield the expression plasmid pET28arATB. For the convenience of detection and following purification, expression vector encoded recombinant protein fused with vector-encoded His-tag peptide at the N-terminal site.

### 2.4. Expression of B-chains in *E. coli*

The rATB was expressed in *E. coli* BL21. An overnight culture of each fresh transformant was diluted 1 : 100 in 0.5 L of fresh Luria-Bertani medium (LB) containing kanamycin (30 *μ*g·mL^−1^) and incubated at 30°C to an OD_600_ of 0.6-0.7. Isopropyl *β*-D-thiogalactoside (1.0 mM) was added and culture incubated for an additional 3-4 h to induce expression. Cells were then harvested by centrifuge at 4000 g; resuspended in phosphate-buffered saline (PBS) at pH 7.4, and disrupted by sonication. Cell lysate was centrifuged at 20,000 g, pellet and supernatant were each analyzed by SDS-PAGE to detect rATB.

### 2.5. Isolation of rATB from Insoluble Inclusion Bodies

The pellet from lysate of cells transformed with pET-28a rATB was washed exhaustively with PBS containing 1% Triton X-100 to remove adsorbed proteins; rATB was then subjected to refolding protocol.

### 2.6. Refolding of rATB

Refolding process was based on modified protocol as previously described [11]. After isolation of rATB from inclusion bodies, insoluble protein (2 mg/mL) was dissolved in 2.5 mL of solubilization buffer (8 M urea, 10 mM NaCl, 10 mM Tris-HCl, pH 8.5). The same volume of reduction buffer (50 mM DTT in 50 mM Tris-HCl, pH 8.5) was added dropwise to the sample, and the mixture was incubated at room temperature for 2 h. The solution was then diluted with oxidation buffer (4 M urea, 5 mM cysteine, 1 mM cystine, 5 mM d-Gal, 50 mM Tris-Cl, pH 8.5) and dialyzed against the same buffer containing half the urea concentration of the previous round. During this process, we changed the dialysis buffer every 12 h. After complete removal of urea, solution was concentrated by dialofiltration in a Centriprep 10 apparatus (Amicon). Circular dichroism (CD) measurements and hemagglutination activity were used to check extent of refolding. For biological assays, soluble rATB (0.5 mg/mL) was stored in 2.2% glycine buffer, pH 7.4 to maintain its activity. Level of contaminating lipopolysaccharide (LPS) in purified protein was determined by Limulus Amebocyte Lysate Assay (Wako Chemicals USA, Inc.), and the final concentration of LPS (<5 ng/mg protein) added to cell cultures was never more than 25 pg/mL.

### 2.7. CD Measurements

CD spectra were recorded with an Aviv 202 SF CD spectrometer (Lakewood, NJ, USA) from 195 to 250 nm in quartz cuvettes of 1 mm path length, recorded as an average of three scans. CD spectra were measured in protein solutions of about 0.05 mg/mL in 20 mM phosphate buffer, and pH 7.4. CD spectra were obtained in millidegrees and converted to molar ellipticity. Secondary structure content was estimated from the CD spectra by CONTIN, SELCON, and CDSSTR methods [12].

### 2.8. Hemagglutination Activity Assay

Hemagglutination activity was measured in a 96-well microtiter plate using a 2% suspension of human erythrocytes. Each well of the microtiter plate contained 50 *μ*L of 2% erythrocyte suspension and 50 *μ*L of serially diluted rATB. The activity was evaluated by visualizing the minimum amount of protein causing agglutination. For hemagglutination inhibition assay, 50 *μ*L of 0.2 M sugar solution was added to each well to test its ability to inhibit hemagglutination activity of rATB. Serially diluted Gal and GalNAc solutions were used to determine concentration needed for complete inhibition of rATB.

### 2.9. Molecular Modeling and Docking

Articulatin B-chain structure was modeled from the template EML-I (PDB ID: **1OQL**) using the program Accelry-MODEL. Ligand and protein were prepared using program sybyl7.3, preliminary docking obtained by GOLD 3.1.1 program. For precise docking, preliminary docking data were further processed via Glide. Hydrogen bond interactions between protein and ligand were drawn using PyMoL.

### 2.10. Blood Donors

All samples were collected from healthy volunteers. The Ethical Committee of China Medical University approved the present study, with informed consent obtained from all participants.

### 2.11. Preparation of Lysed Whole Blood Cells for rATB Binding Assay and Immunoflowcytometric Analysis

The heparinized whole blood cells were treated with AKC lysis buffer to remove red blood cells, then washed twice and resuspended in PBS pH 7.4. These lysed whole blood cells, consisting of leukocytes (1 × 10^6^ cells per mL), were incubated with 500 ng/mL rATB in the presence or absence of 50 mM Gal or Man at 4°C for 30 minutes, followed by immunoflowcytometric analysis with a FACSCanto Flow cytometer (BD Biosciences). A mouse anti-His-tag monoclonal antibody (R&D) which targeted the N-terminal His-tag of rATB was used as primary antibody, rabbit antimouse antibody labeled with PE (R&D) as secondary antibody. The rATB binding to leukocytes was determined by detecting intensity of fluorescence on different leukocyte populations defined by setting a polygonal gate in forward/sideward scatter dot plot. These results were analyzed with CellQuest software (BD Biosciences).

### 2.12. Preparation of Peripheral Blood Mononuclear Cells and Cytokine Induction Experiments

PBMCs were isolated from whole blood by centrifugation through Ficoll-Hypaque solution (Histopaque-1077) and then resuspended in RPMI 1640 medium (Gibco) supplemented with 10% FCS. PBMCs (5 × 10^5^ cells/mL, 1 mL/well) were treated with rATB (0.8 ng/mL–5 *μ*g/mL) or vehicle buffer (2.2% glycine buffer) or LPS (25 *μ*g/mL, Sigma) in the presence or absence of Gal or Man, cultured in 24-well tissue plates. After 72 h of culturing, supernatants were harvested and tested for production of TNF-*α* and IL-6 using Human Cytokine Kit (BD Biosciences). Viability was assessed in parallel in each experiment with CellTiter 96 AQueous kit (Promega). Experiments with polymyxin B (Calbiochem) to block *in vitro* effects of endotoxin were carried out by preincubation of rATB with 5 mg/mL polymyxin B for l h at room temperature.

### 2.13. Statistical Analysis

Data are expressed as means ± s.d. Comparisons were made by two-tailed *t*-tests; significance was accepted at 0.05 level of probability (*P* < .05).

## 3. Results

### 3.1. Sequence Identification and Analysis of the Articulatin B-Chain Gene

**Figure 1:**
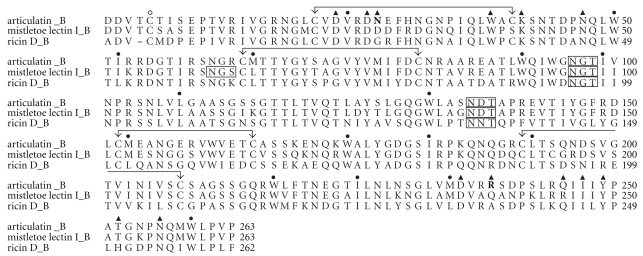
Alignment of deduced amino acid sequence of articulatin B-chain (GeneBank accession no. EF620539), ML-I B-chain (A58957), and ricin D B-chain (P02879). Residues participating in N- and C-terminal sugar binding sites of the B-chain are indicated by ▲ [9, 10]; ● denotes highly conserved residues forming a hydrophobic core of B-chain domains. Residues participating in sugar binding sites, but distinct from the conserved residues, are in boldface type. Joined arrows mark Cys residues forming intrachain disulfide bonds. Cys residues forming interchain disulfide bond are marked with ∘. Potential *N*-glycosylation sites are boxed.

Whole gene encoding for type-2 RIP from *Viscum articulatum* was previously cloned as described in Section 2 and designated the *articulatin* gene. Based on alignment with other related type-2 RIP genes, we delimited gene fragment coding for B-chain. The B-chain coding sequence of 789 bp (263 amino acid residues with a calculated *M*_*r*_ of 28,877) was amplified by PCR from genomic DNA of *V. articulatum* for sequence determination and heterologous gene expression. Deduced amino acid sequences of articulatin B-chain had an expected high overall sequence identity level to ML-I B-chain (81%). Alignment of articulatin sequences, ML-I, and ricin D showed amino acid residues responsible for sugar binding in ML-I B-chain and ricin B-chain as conserved or replaced by related residues in articulatin B-chain (Figure 1).

### 3.2. Molecular Modeling and Docking Experiments

**Figure 2:**
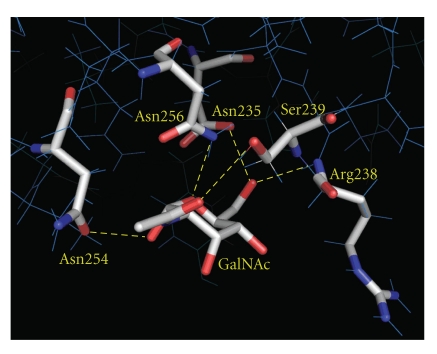
Best glide docking result of GalNAc in C-terminal sugar binding site of articulatin B-chain. GalNAc binding residues and GalNAc appear as stick models; red: oxygen; blue: nitrogen; gray: carbon; yellow dashed lines: hydrogen bonds.

Due to inadequate identification of native articulatin and fear that articulatin has an inactive B-chain as previously described (SNLRP (*Sambucus nigra *lectin-related protein), a novel type-2 RIP with an inactive B-chain from *Sambucus nigra*) [13], we performed molecular modeling and docking to examine postulated structure of articulatin B-chain before rATB expression. The 3D model of the articulatin B-chain exhibited overall folding similar to that of ML-I B-chain used as template. Simulated structure contained an N- and a C-terminal carbohydrate binding site. In the best glide-docking of GalNAc at the C-terminal sugar binding site of articulatin B-chain, five amino acid residues—Asp235, Arg238, Ser239, Asn254, and Asn256—can directly donate hydrogen bonds to GalNAc (Figure 2), arguing potential carbohydrate binding capacity of articulatin B-chain with monosaccharide-binding specificity toward galactoside moiety.

### 3.3. Expression and Refolding of Recombinant Articulatin B-Chain

**Figure 3:**
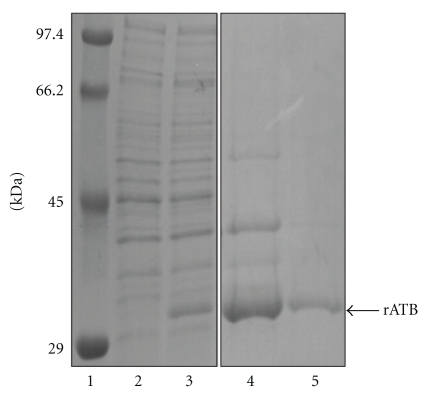
SDS-PAGE (12.5% polyacrylamide gel) of rATB. Lanes: 1: molecular size markers; 2: *E. coli* BL21 (pET28arATB) prior to induction; 3: *E. coli* BL21 (pET28arATB) after induction; 4: 5 *μ*g denatured rATB in inclusion bodies; 5: 1 *μ*g refolded soluble rATB.

In light of the potential lectin activity of articulatin B-chain suggested by modeling and docking, we decided to create active rATB to scrutinize potential uses of the B-chain fragment. We cloned articulatin B-chain gene fragment and constructed plasmid pET28arATB for expressing rATB in *E. coli* BL21, as described in Section 2. Expressed rATB was totally sequestered in the inclusion bodies fraction. Induction under various conditions failed to increase solubility of rATB; however, production of proteins as inclusion bodies offered the advantage of easy purification. Thus, rATB was purified by washing the inclusion body fraction with PBS containing 1% Triton X-100. This process may allow the isolation of inclusion bodies from other cell components [14]. Then, these washed inclusion bodies were solubilized in 8 M urea and submitted to the refolding process in the presence of redox-pair and galactose. Yield of refolded rATB was about 32 mg/L culture. SDS-PAGE showed the molecular mass of rATB as approximately 30 kDa (Figure 3).

### 3.4. Stabilization and Monitoring of Soluble Recombinant Articulatin B-Chain

Previous study has shown that soluble recombinant ricin B-chain expressed in *E. coli*, which although initially soluble and biologically active, was unstable and tended to aggregate rapidly [15]. Similarly, we here observed that soluble refolded rATB gradually aggregated and precipitated during dialysis against phosphate buffer saline. We further discovered that the aggregation was enhanced in the presence of NaCl, which contributed to high ionic strength. Thus, we here developed isotonic solution of low ionic strength, 2.2% glycine buffer, to keep soluble refolded rATB stable and active for subsequent functional assays.

**Figure 4:**
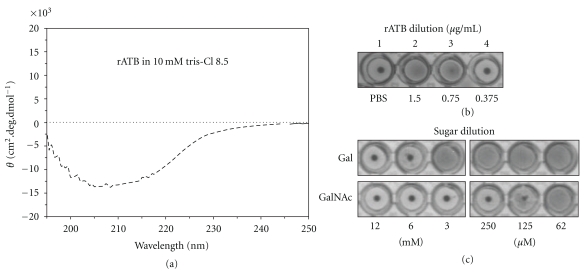
Monitoring of soluble refolded ATB. (a) Circular dichroism (CD) spectra of rATB after refolding. Protein concentration: 0.05 mg/mL. (b) Hemagglutination activity of rATB. Wells: 1 PBS, negative control; 2–4 serially diluted refolded rATB. (c) rATB hemagglutination inhibition assay. Serially diluted Gal and GalNAc determined concentration needed for total inhibition of 30 *μ*g/mL rATB.

CD analysis was used to monitor protein refolding. Deconvolution of far-UV CD spectra of refolded rATB (Figure 4(a)) yielded approximately 20% *α*-helix and 27% *β*-sheet for rATB. Existence of two potential carbohydrate binding sites led us to perform hemagglutination (HA) and test rATB lectin activity. Minimum concentration required for rATB to achieve hemagglutination was around 0.75 *μ*g·mL^−1^ (Figure 4(b)). Hemagglutination test in the presence of various sugars (hemagglutination inhibition assay) further showed binding specificity of rATB; D-Gal and GalNAC completely inhibited rATB at 12 mM and 250 *μ*M, respectively, while D-glucose and D-mannose showed no interaction with rATB, even at 0.2 M (Figure 4(c)). Of note, the lectin activity of rATB remained stable at 4°C in glycine buffer for over one week, whereas rATB gradually lost its activity in 24 h while kept in PBS. These results, consistent with the predicted lectin activity from modeling and docking experiments, revealed stabilized rATB available for future biological studies.

### 3.5. Biological Activities of rATB on PBMCs

**Figure 5:**
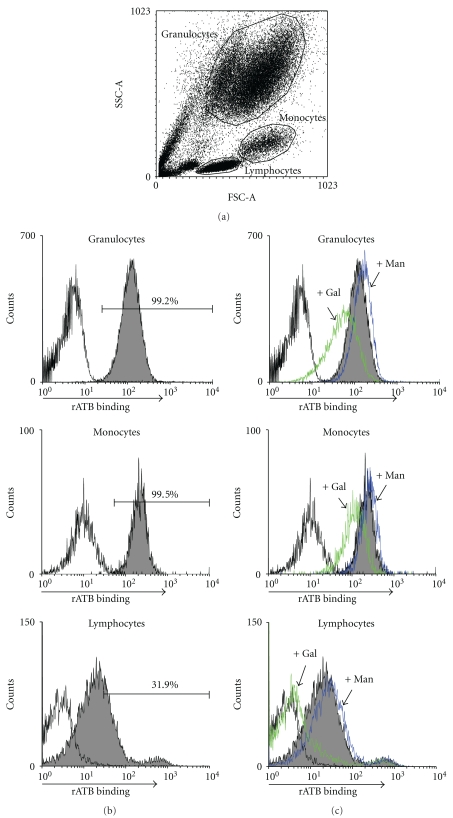
Binding capability of rATB to diverse leukocytes. Flow cytometric analysis of lysed whole blood after incubation with rATB (500 ng/mL) in the presence or absence of sugars (50 mM). (a) Granulocytes, monocytes, and lymphocytes were identified by setting a polygonal gate in a forward scatter/sideward scatter dot plot. (b) Percentages of positively stained granulocytes, monocytes, and lymphocytes exhibit a shift toward the right in comparison with control. (c) Leukocytes were preincubated with competitive sugar (Gal) or uncompetitive sugar (Man) as indicated by arrows. Data from one of three independent experiments with similar results are shown.

Stabilization of rATB allowed us to probe its interactions with immune cells. Leukocytes from lysed whole blood of healthy volunteers were incubated with rATB *in vitro*, then analyzed by immunoflowcytometry. Granulocytes, lymphocytes, and monocytes identified by setting a polygonal gate in a forward scatter/sideward scatter dot plot (Figure 5(a)) all showed binding affinity for rATB, whereas granulocytes and monocytes had higher affinity than lymphocytes (Figure 5(b)). Presence of the binding inhibitor of rATB, D-galactose, significantly reduced these bindings (Figure 5(c)).

**Figure 6:**
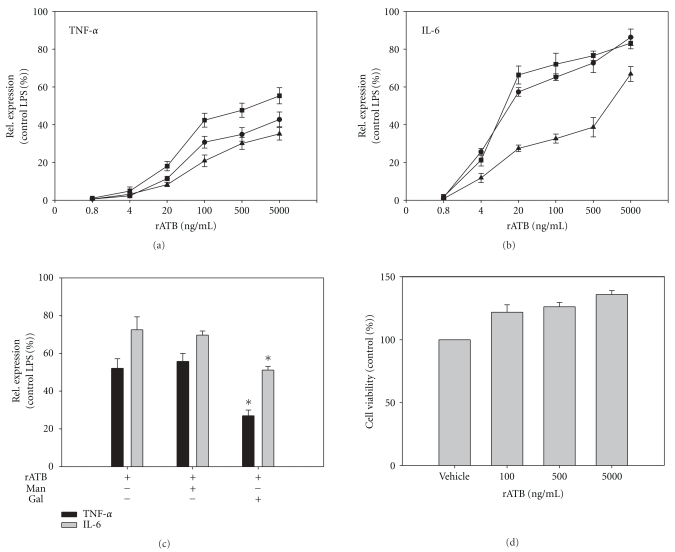
Biological effects of rATB on PBMCs. Relative concentration of TNF-*α* (a) and IL-6 (b) in culture of PBMCs (5 × 10^5^/mL) treated with rATB (0.8–5000 ng/mL). Data from three individual donors are shown. Measurements were performed in triplicate. Means ± s.d. are shown. (c) Relative concentration of TNF-*α* and IL-6 in culture of PBMCs (5 × 10^5^/mL), preincubated with Gal (50 mM), Man (50 mM), or vehicle buffer before rATB (500 ng/mL) treatment. Values shown are averages ± s.d. of one experiment assayed in triplicate. *P < .05 versus vehicle control. (d) Viability of PBMCs treated with 100–5000 ng/mL rATB. The cell viability with vehicle buffer treatment is set as 100%. Values are expressed as the mean of three replicates ± s.d.

We next explored whether rATB could act as an immunomodulator. We isolated PBMCs from healthy volunteers and *in vitro* treated with rATB. In this *in vitro* culture system, rATB dose-dependently stimulated PBMCs to release TNF-*α* (Figure 6(a)) and IL-6 (Figure 6(b)). Competitive (Gal) but not uncompetitive sugar (Man) significantly reduced cytokine release induced by rATB (Figure 6(c)). In parallel, we performed cell survival assay in each experiment to evaluate cytotoxicity of rATB. Even at maximum applied concentration, no obvious cytotoxicity of rATB was observed (Figure 6(d)).

Since endotoxin can display similar cytokine inducing effects, we examined LPS contamination in rATB preparation using commercially available Limulus Amebocyte Lysate Kit. In the cytokine induction experiment, LPS contamination from rATB was lower than 25 pg/mL, which had no effect on this system. Moreover, the presence of polymyxin B (5 *μ*g/mL), an LPS *in vitro* neutralizer, also had no influence on release of cytokines induced by rATB.

## 4. Discussion

This paper investigates biological functions of new recombinant type-2 RIP B-chain, rATB. Lectin activity of rATB resembles that of other type-2 RIPs: for example, it can agglutinate red blood cells. This means that it has at least two binding domains, with rATB binding specificity to monosaccharide toward galactoside moiety. In particular, we find preferential binding of rATB to monocytes and granulocytes similar to that of ML-I [16]; rATB can also increase cytokine secretion from PBMCs *in vitro*. To the best of our knowledge, this is the first report to show nonglycosylated recombinant B-chain of type-2 RIP capable of inducing cytokine release from PBMCs. This finding is consistent with studies of isolated mistletoe lectin B-chain on immunostimulation [6].

Sequence analysis shows articulatin B-chain containing two *N*-glycosylation sites. As scarcity of the plant makes it impossible to obtain enough native protein for analysis, we currently are unable to clarify the biological role of protein glycosylation. Prior studies showed *N*-glycosylation as important for stability and lectin activity of a ricin B-chain. Nonglycosylated mutant ricin B-chain rapidly aggregates; even when it appears completely soluble, it is devoid of sugar binding activity [17, 18]. Similarly, we found nonglycosylated rATB gradually aggregating and losing lectin activity while stored in solutions of high ionic strength. For this study we thus developed a solution of low ionic strength for rATB storage to stabilize activity of rATB. Such long-term storage stability is obviously critical for research on rATB and other nonglycosylated recombinant type-2 RIP B-chains. Interestingly, rATB is still active in our *in vitro* experiments, where high ionic strength usually exists. This supposes that rATB can reach its target in time; effect of ligand binding might also stabilize the protein structure for functioning, as previously described in [19].

Experiments in animal models suggest that ML-I-mediated inhibition of tumor growth is associated with immunomodulatory efficacy [20]. In a Scid mouse xenograft model, Thies et al. recently reported low-dose ML-I reducing melanoma growth and number of metastasis primarily due to its immunomodulatory effects, while higher dose lost this effect. One possible mechanism might be that low-dose mistletoe lectin I increased the number of infiltrating dendritic cells, which can lead to apoptosis of tumor cells; at a higher dose, ML-I induced apoptosis of dendritic cells and thus lost its antitumor effect [21]. Based on this observation, it appears that high cytotoxicity of ML-I might have a negative effect on stimulating immune cells. In this study, rATB lacks the toxic A-chain and yet retains immunostimulating capability without obvious cytotoxicity, suggesting that this active recombinant B-chain, instead of heterodimer, might be applied in treatment at a higher dose to achieve substantial enhancement of immune response without unwanted cytotoxicity. In this respect, rATB merits further investigation to ascertain immunomodulatory efficacy and might offer the advantage of a controlled clinically beneficial immunomodulation in cancer treatment. In sum, cloning, heterologous expression, refolding, and long-term storage of rATB have been effectively established. Likewise, rATB can bind leukocytes and stimulate PBMCs to release cytokines. Results portend such B-chain fragment as an immunomodulator.

## Abbreviations

RIP: Ribosome-inactivating protein
rATB: Recombinant articulatin B-chain
ML: Mistletoe lectin
Gal: Galactose
GalNAc: *N*-acetylgalactosamine
PBMCs: Peripheral blood mononuclear cells.
